# Improving Fast Pyrolysis Bio-Oil Yield and Quality by Alkali Removal from Feedstock

**DOI:** 10.1021/acs.energyfuels.1c04331

**Published:** 2022-03-29

**Authors:** Elmeri Pienihäkkinen, Christian Lindfors, Taina Ohra-aho, Anja Oasmaa

**Affiliations:** VTT Technical Research Center of Finland Ltd., P.O. Box 1000, FI-02044 Espoo, Finland

## Abstract

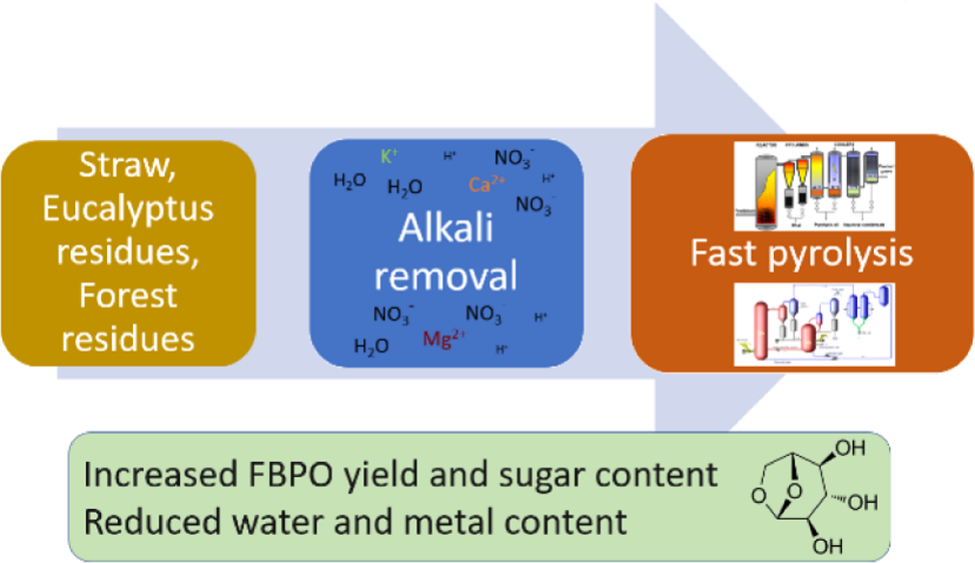

Alkali removal from forest residues, eucalyptus residues, and wheat straw was studied by water and dilute nitric acid leaching. Leaching parameters were optimized for each feedstock in laboratory-scale experiments. After the optimization of leaching on the laboratory scale, nitric acid-leached and untreated feedstocks were pyrolyzed in a bench-scale bubbling fluidized bed unit. In the case of eucalyptus residues and wheat straw, nitric acid leaching was found to increase the organic liquid yield compared to untreated feedstock. In addition, the sugar content of the fast pyrolysis bio-oils was increased, and the alkali content reduced. On the other hand, the pyrolysis experiments with acid-leached forest residues were unsuccessful due to the bed agglomeration. These problems are expected to be a result of the lack of catalytically active elements in biomass which enhance especially the cracking reactions of lignin. Finally, the results were demonstrated in the pilot-scale unit where nitric acid-leached oat straw was pyrolyzed with high organic liquid yield.

## 1 Introduction

Increasing pressure to reduce greenhouse gas emissions has led to growing interest toward sustainable biofuels and bio-based chemicals. Fast pyrolysis of lignocellulosic feedstocks is one of the routes to produce liquid products suitable for these purposes, and utilization of non-edible lignocellulosic waste streams as feedstock is one of the most promising ways to maximize the emission reductions.^1^ Utilization of wastes is as well a great way to expand the feedstock pool for fast pyrolysis where rather pure wood, such as saw dust or forest residues, is the dominant feedstock at the industrial scale. However, low-quality waste streams with high concentrations of impurities are problematic from the perspective of biomass pyrolysis and introduce the need for feedstock pretreatment.

Main impurities present in lignocellulosic feedstocks are inorganic elements of ash. Components of ash, especially alkali and alkaline earth metals (AAEMs) such as K, Na, Ca, and Mg, are catalytically active during pyrolysis.^2,3^ Removal of biomass inorganics prior to thermochemical processes (combustion, gasification, and pyrolysis) has been studied already in the 80s and 90s.^2,4−6^ In addition, metal removal from feedstock of the pulping industry has been studied.^7^ Main methods are based on the water or acid leaching of biomass, which are gentle toward the structure of biomass but still efficient in inorganic removal.^8^ Several studies indicate that approximately 80–90% of AAEMs in biomasses are in the water- or acid-soluble form,^3,9−12^ K and Na being easier to remove with water than Mg and especially Ca.^9^ This is logical due to the higher mobilities and different binding strengths of monovalent K^+^ and Na^+^ ions compared to those of divalent Mg^2+^ and Ca^2+^ ions.^13^ Water-soluble (WS) inorganics are mainly WS salts and free ions in fluid matter of plants. The acid-soluble part can include not only salts and minerals only soluble in acids but also WS cations trapped in the ion-exchange matrix of biomass. The ion-exchange capacity of plant fiber is expected to result from the presence of carboxylic acid groups in polysaccharides, mainly in hemicelluloses and in pectin substances.^7^ The completely insoluble part of AAEMs might be insoluble salts or species strongly bound to organic molecules of biomass.^9,14^

Jensen et al.^2^ reported that pyrolysis of leached wheat straw, with (TG–FTIR) thermogravimetric-Fourier transform infrared spectroscopy, increased bio-oil formation from 32 to 64 wt % and reduced char formation from 20 to 12 wt %, compared to the pyrolysis of untreated wheat straw. Piskorz et al.^5^ reported not only significant improvement in fast pyrolysis bio-oil (FPBO) yield but also altered chemical composition of produced liquids. After these experiments, leaching of inorganics from fast pyrolysis feedstock has awaken a lot of research interest, and a couple of comprehensive review articles have been written.^8,15^

These early findings have later been confirmed, and now it is well known that catalytic activity of ash results in decreased FPBO yield and altered chemical composition. Organic liquid yields are higher with low-ash than high-ash feedstocks,^16^ and de-ashed feedstocks have been reported to give higher FPBO yields and a lower water content in FPBOs.^11,17,18^ In addition to yield losses, significant differences are seen in the chemical composition of FPBOs. Stefanidis et al.^19^ and Mourant et al.^11^ compared the chemical composition of FPBOs produced from de-ashed and untreated feedstocks. Sugar concentrations of liquids were significantly lower when inorganic cations were present in biomass. On the other hand, concentrations of C=O compounds, such as furans and ketones, were higher when cations were present. Authors postulated that this might indicate that metals can catalyze the homolysis of pyranose rings to carbonyl compounds at the expense of anhydrosugar formation.^19^ Potassium is presumed to be the most active element in catalyzing these competitive degradation reactions of polysaccharide derivatives.^20,21^

Regarding the effects on the lignin fraction of biomass, Oasmaa et al.^22^ reported that lignin was cracked more with high-ash feedstocks and that FPBO produced included less high-molecular weight (HMW) lignin. Stefanidis et al.^19^ presented comparable results and reported that de-ashed feedstocks resulted in lower concentrations of phenolic compounds. Authors concluded that the calcium concentration was the most significant parameter regarding the lignin cracking reactions. These results indicate that different elements are active in different reactions. Thus, the concentrations of inorganic elements are much more important than the total ash content of feedstock.

In addition to effects presented above, a high ash content of feedstock typically increases the inorganic content of produced FPBO, which may be problematic from the perspective of further refining.^23^ Leijenhorst et al.^24^ reported that although AAEMs were predominantly retained in char, a significant amount was also transferred into FPBO. Transfer rates of AAEMs were not equal. A larger portion of potassium and sodium compared to that of calcium and magnesium transferred into FPBO. Different solubilities of AAEM salts into FPBO can also affect this. The average transfer rate of potassium and sodium was 8% and with calcium and magnesium 2%.^24^

AAEMs have also been reported to accumulate on the acidic zeolite catalyst used in catalytic fast pyrolysis. Accumulation has been connected with the deactivation of the catalyst, and accumulated inorganics, especially potassium, might change the behavior of the catalyst and its activity.^25,26^ Similar catalysts can also be used in upgrading of FPBO.^23^ AAEMs might also catalyze the aging reactions of pyrolysis liquids during storage.^27^ For all the above-mentioned reasons, removal of alkali metals prior to pyrolysis has awaken a lot of research interest.

The objective of this study was to improve the carbon efficiency of the total biofuel chain and to demonstrate the effects of pretreatment on bench- and pilot-scale fast pyrolysis. This was done by improving organic liquid yields of the pyrolysis process by removing the AAEMs from feedstock. Removal of alkalis was studied with three industrially relevant feedstocks (forest residues, eucalyptus residues, and wheat straw). Studied biomass pretreatment methods to remove alkalis were water and acid leaching. First, leaching parameters were optimized in laboratory-scale experiments after which a suitable amount of feedstock was prepared for the bench-scale fluidized bed pyrolysis tests. Untreated and de-ashed feedstocks were pyrolyzed, and mass balances and liquid properties were analyzed and compared. In addition, AAEM contents of the FPBOs were measured to clarify their fate after leaching. Finally, the pilot experiment was conducted with leached oat straw to demonstrate and confirm the effects of feedstock pretreatment on a larger scale. To our knowledge, no previous published work with leached feedstocks has been carried out at this scale, with circulating fluidized bed systems.

## 2 Materials and Methods

### 2.1 Materials

Feedstocks for laboratory- and bench-scale leaching experiments were pine forest residues, eucalyptus residues, and wheat straw. All feedstocks were provided as dried to a moisture content of 6–10 w %. Feedstocks were ground and sieved to particle size 0.55–0.98 mm prior to leaching. Deionized water (DI) was used as leaching liquid in the experiments with pure water, but in acid leaching, also tap water was tested. Acidic leaching liquids were diluted from 65 wt % nitric acid.

Feedstocks used on the pilot scale were wheat straw and oat straw. Leaching pretreatment was done for the oat straw. Prior to leaching, oat straw was crushed to particle size < 4 mm. After leaching, straw was dried, pelletized, and ground to a particle size of 0.5–3.0 mm, which is suitable size for the VTT pyrolysis pilot unit. Wheat straw was not leached, but it was pelletized and ground in the similar way as the leached oat straw.

### 2.2 Leaching Procedures

Laboratory-scale leaching experiments were carried out in glass bottles at atmospheric pressure. Biomass and preheated leaching liquid were weighted in the bottle, and the suspension was stirred continuously with a magnet stirrer. Temperature of the suspension was followed with a thermometer. Bottles were merged into an oil bath, and the oil was heated with the plate heater. After the aimed residence time was reached, the sample was filtered with a Buchner funnel and rinsed with de-ionized water. The leached biomass sample was collected, weighted, and its moisture content was analyzed. Solid samples were dried at 80 °C overnight for further analyses. To ensure the results and minimize the possible heterogeneity of solid samples, all tests were carried out as duplicates.

Bench-scale leaching experiments were performed in a cylinder-shaped and Teflon-coated vessel equipped with a heating jacket and mixer. Volume of the vessel was 100 L. Leaching conditions for bench-scale tests were chosen based on the results from laboratory experiments and are presented in Table 1. Leaching liquid was loaded into the vessel, and when the correct temperature was reached, 5 kg of feedstock was weighted into the vessel. The suspension was mixed, and temperature of the suspension was followed with a thermometer. After the residence time was reached, the suspension was drained from the valve located at the bottom of the vessel. Then, the suspension was filtered, and filtered solids were rinsed with de-ionized water. After rinsing, biomass was dried overnight in the oven at 50 °C to reach the aimed 5–10% moisture content.

**Table 1:** Leaching Parameters on Pilot and Bench Scale^a^

sample	temp. (°C)	time (min)	acid concentration (wt %)	leaching liquid (B/LL)	rinsing water (B/W)
forest residues for bench scale	50	30	1	1:10	1:10
eucalyptus residues for bench scale	50	30	1	1:10	1:10
wheat straw for bench scale	20	30	0.5	1:10	1:10
oat straw for pilot scale	20	30	0.5	1:20	1:20

a B = biomass, LL = leaching liquid, W = water.

The leaching procedure for the pilot-scale study followed same principles as in laboratory- and bench-scale experiments. Straw and leaching liquid were mixed in a large vessel, and after the residence time was reached, the suspension was pumped to filter screw press, where water and solids were separated. Due to the equipment limitations, a larger amount of leaching liquid was used. Consistency, that is, the dry matter content of the liquid–straw suspension, was limited to 5 w % to ensure proper mixing and pumping of the suspension. This corresponds to the biomass to leaching liquid ratio (B/LL) of 1:20. Leaching was conducted in several batches. After the leaching, feedstock was dried for further pelletizing and grinding.

### 2.3 Pyrolysis Experiments at Bench Scale

**Figure 1:**
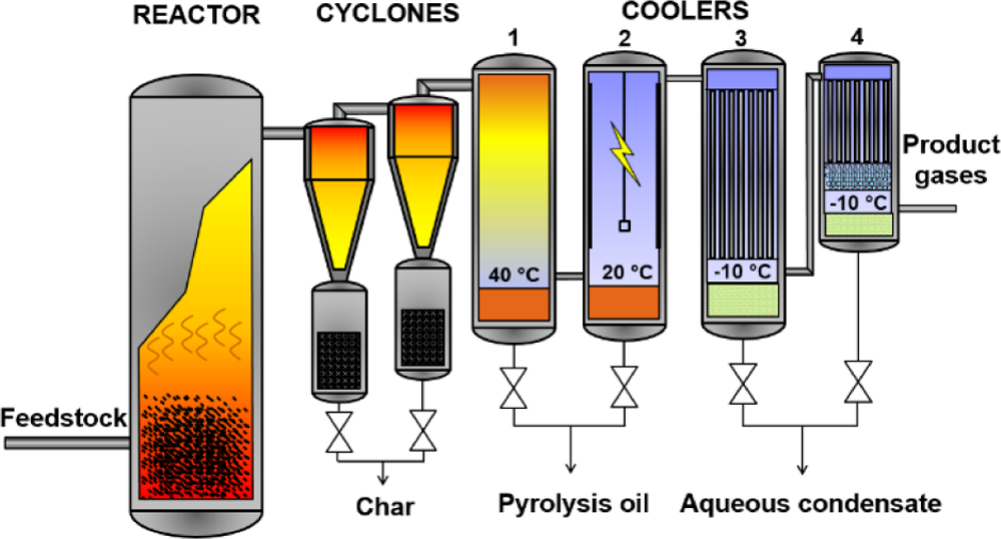
Schematic flow diagram of the bench-scale fast pyrolysis BFB unit. Product condensing consists of four coolers: (1) water-cooled heat exchanger, (2) electrostatic precipitator, (3) glycol-cooled tube heat exchanger, and (4) smaller glycol-cooled tube heat exchanger with glass packings.

Bench-scale pyrolysis experiments were carried out in a bubbling fluidized bed (BFB) reactor using nitrogen as fluidization medium (Figure 1). Detailed description of equipment and the procedure is presented elsewhere.^28^ The reactor was operated at atmospheric pressure, and temperature used was 480 °C. Temperature varied ±5 °C during the experiments, and the temperature profile was uniform through the whole reactor length.

Fluidization nitrogen was fed into the reactor through a gas distributor plate located at the reactor bottom. The fluidization gas flow rate was adjusted so that the superficial gas phase residence time under the reactor conditions was 1 s. In reality, the residence time will be shorter due to the evolution of gases and vapors from the feedstock. In the reactor, 300 g of white aluminum oxide (0.56–0.71 mm, ρ = 4 000 g/dm^3^) was used as bed material during the experiments. The feedstock feed rate was calibrated to 800 g/h.

The char was separated from the pyrolysis gases with two cyclones. After the cyclones, hot vapors and gases were first cooled indirectly with cold water in a water-cooled heat exchanger (40 °C) after which vapors and gases were passed to an electrostatic precipitator (20 °C). From the electrostatic precipitator, the non-condensed water and light organics were led to two glycol coolers (−10 °C); one tube heat exchanger and a second smaller tube heat exchanger filled with additional glass packings. The composition of the non-condensable gases was analyzed by micro-GC (gas chromatography). Product yields are reported on the dry basis of the starting feedstock.

### 2.4 Pyrolysis Experiments at Pilot Scale

In the VTT’s CFB pilot, the ground and sieved raw material was fed into the reactor with a screw feeder. The design feed capacity for the unit is 20 kg/h for dried biomass feedstocks. The reactor was a circulated fluidized bed operated at atmospheric pressure and heated with the hot sand from the combustor. Sand used was quartz sand (0.1–0.6 mm, ρ = 2 600 g/dm^3^). Raw material was introduced into the cold section of the riser reactor with the cold fluidization gas coming from the reactor bottom, after which raw material particles were carried upward to come in contact with the hot sand. Hot sand was fed into the reactor with a screw feeder, and the reactor temperature was controlled with the sand flow rate. After the introduction of hot sand, the mixture of solids was carried through the reactor to the cyclones. During this time, the majority of pyrolysis reactions took place. The planned pyrolysis temperatures were 480, 490, and 500 °C, and the superficial fluidization gas velocity was 7 m/s. The main part of the char particles and heat transfer sand were removed by two cyclones from the hot product gases and vapors before entering the liquid recovery system.

**Figure 2:**
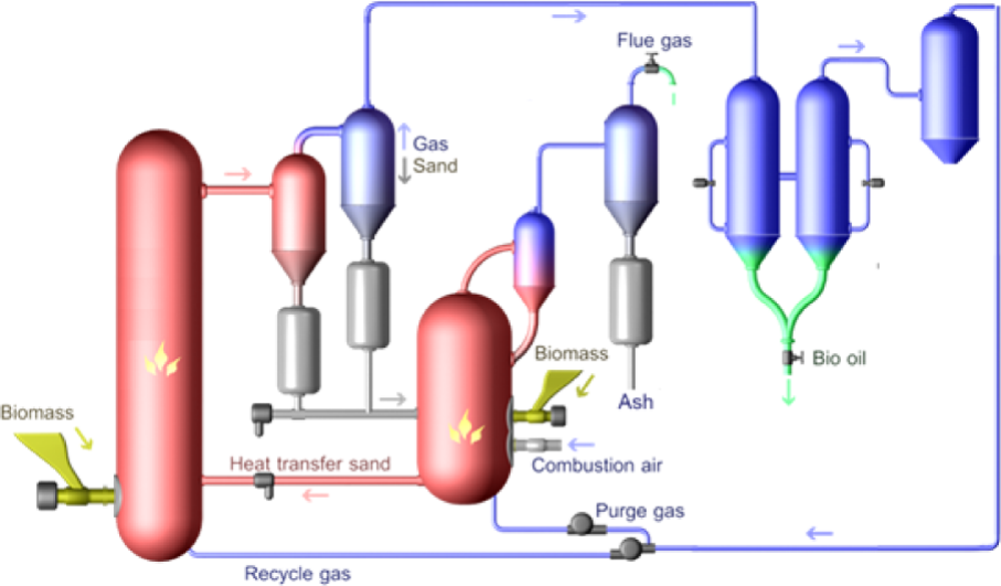
Schematic representation of the VTT’s CFB pilot.

In the liquid recovery system, two scrubbers and one cooler were used. The vapors were condensed by using the pyrolysis liquid as a cooling agent. The temperature of the scrubbers was kept at 40 °C. A part of the non-condensable gases was used for fluidization, and the rest was burned in the combustor. Most of the ash from the feedstock ends with the char in the combustor. The combustor was operated as a BFB, and the temperature was controlled to 670–700 °C by feeding ground pellets into it. After the combustor, one cyclone and a hot gas filter were used to remove the fine dust and fly ash from the flue gases. Before the hot gas filter, flue gas was cooled to <250 °C using a tube heat exchanger and water quench. A schematic flow diagram of the pilot unit is presented in Figure 2. Product yields are reported on the dry basis of the starting feedstock.

### 2.5 Characterization Methods

The ash and moisture content of untreated and leached biomass samples was determined by thermogravimetric analysis according to standard SFS-EN ISO 18122. The equipment used was the LECO Corporation TGA-601 Thermo Gravimetric Analyzer. The moisture content of feedstocks was followed daily with an Adam PMB Moisture Analyzer. Elemental analyses of inorganics in biomass were measured by (IC) ion chromatography and (ICP-OES) inductively coupled plasma optical emission spectrometry methods according to standards SFS-EN ISO 10304-1 and SFS-EN ISO 11885, respectively. By IC, Cl and S and by ICP-OES, Ca, Mg, Na, K, P, Fe, Al, and Si were analyzed.

Physical characterization of the FPBO was carried out by employing modified standard methods.^29^ The water content was analyzed by Karl Fischer titration using a Metrohm 795 KFT Titrino titrator (ASTM E 203). Elemental composition (CHN) was analyzed using an Elementar VARIOMAX CHN analyzer (ASTM D 5291), and a higher heating value (HHV) was measured using an IKA Werke C 5000 Control calorimeter (DIN 51900). The total acid number (TAN) was determined with a 785 DMP Titrino analyzer (ASTM D 664), and the micro carbon residue (MCR) was determined using an Alcor Micro Carbon Residue Tester (ASTM D 4530). The ash content of the liquid was determined by combusting the residue from the MCR determination in a muffle furnace at 775 °C. The inorganic content of the liquids was analyzed according to standards DIN 51727 B:2011 (Cl) and DIN EN ISO 11885:2009 (other inorganics).

The chemical composition of the FPBOs was determined with the solvent fractionation scheme. In this method, the FPBO is first divided into a WS and a water-insoluble (WIS) fraction by water extraction. The WS fraction is further extracted with diethyl ether to an ether-soluble (ES) and an ether-insoluble (EIS, sugar-like material) fraction. The water-insoluble fraction is extracted with dichloromethane (DCM) to a DCM-soluble fraction containing low-molecular weight (LMW) lignin and a DCM-insoluble fraction containing HMW lignin. In general, the LMW fraction contains poorly WS lignin monomers and dimers (MM = 400 Da) and extractives, while the HMW fraction contains powder-like high-molecular mass (MM = 1050 Da) lignin-derived material and solids.^16^

For the carbohydrate and lignin composition, the samples were hydrolyzed with sulfuric acid at two stages, and the resulting monosaccharides were determined by high-performance anion-exchange chromatography (HPAEC) with pulse amperometric detection (Dionex ICS 3000A equipped with a CarboPac PA1 column).^30^ The polysaccharide content in the samples was calculated from the corresponding monosaccharides using an anhydro correction of 0.88 for pentoses and 0.90 for hexoses. The Klason lignin content, that is, the insoluble residue from the hydrolysis, was determined gravimetrically. Acid-soluble lignin was determined from the hydrolysate based on UV spectroscopy at 215 and 280 nm using an equation described by Goldschmid.^31^

## 3 Results and Discussion

### 3.1 Results from Leaching Experiments

#### 3.1.1 Laboratory- and Bench-Scale Experiments

Leaching parameters optimized on the laboratory scale were temperature, residence time, and the amount and acidity of leaching liquid. The total ash content and concentrations of potassium, sodium, calcium, and magnesium were monitored. Data from leaching experiments are presented in the Supporting Information, and only main conclusions are described here. Regarding the parameters of water leaching, no significant changes in the ash removal efficiency were observed when temperature, the amount of leaching liquid, or leaching time was changed. Regarding the amount of liquid, proper mixing of straw was not reached with low amounts of leaching water. Straw is less dense and occupies a larger space and thus needs more liquid to be steadily mixed. The most significant parameter was found to be the acidity of the leaching liquid.

Treatment of wood biomasses in dilute acids was significantly more efficient than treatment in pure water, and increasing the acid concentration improved the leaching. On the other hand, for wheat straw, the differences in total ash removal with acid and water were minor. Acidic leaching liquid was only slightly more efficient, and increasing the acid concentration had no clear effects. One reason for this is expected to be the high silicon content in wheat straw. Silicon is hardly soluble and removable by conventional leaching methods. However, silicon is considered a catalytically inert element during pyrolysis, and its removal is not crucial.^8^ The acid used was nitic acid. Other strong inorganic acids, such as sulfuric acid or hydrochloric acid, have been also proven to be efficient in AAEM removal, but weak acids, such as acetic acid, are only efficient when larger quantities of the acid are used.^32^ Nitric acid was chosen because potential nitrogen traces from the acid were assessed to be less harmful for FPBO quality, compared to, for example, sulfur or chlorine traces. For example, sulfur or chorine can be severe catalyst poisons if further upgrading of the FPBO is considered.^23^ Nitrogen can also be a catalyst poison, but the quantity of nitrogen in biomass and FPBO is naturally much higher, and thus, the potential traces have less severe effects.

Regarding the other studied parameters in acid leaching, increasing temperature was found to enhance the leaching efficiency with wood biomasses. In the case of wheat straw, room temperature was equally efficient. Short leaching time was found to be as efficient as longer time with all feedstocks under acidic conditions. From the scope of the AAEM removal, calcium was the most persistent element. From all feedstocks, potassium and sodium were largely removable by water, although lower concentrations were reached under acidic conditions. Calcium and magnesium were persistent to water treatment but were removed by acid treatment. Removal of calcium was affected the most when parameters were altered. Calcium removal was decreased with a decreasing acid concentration and temperature. Higher temperature can increase the solubility of inorganics and enhance the leaching. The amount of acid is also a crucial aspect if ion-exchangeable cations are to be removed. In principle, only the AAEM content of eucalyptus residues (Table 2) is capable to neutralize 68% of the acid when the biomass to leaching liquid ratio is 1:5, and acid concentrations are 1%. The same value with forest residues is 13%.

**Table 2:** Feedstock Analyses before and after Acid Leaching^a^ for Feedstocks Used in Laboratory- and Bench-Scale Experiments^b^

	unit	forest residues	leached forest residues	eucalyptus residues	leached eucalyptus residues	wheat straw	leached wheat straw
moisture	wt %	5.8	6.7	8.8	3.8	8.0	6.5
ash	wt %, dry	1.2	0.2	4.8	2.8	6.1	4.9
volatiles	wt %, dry ash-free	81.9	86.9	81.6	84.9	80.5	87.5
C	wt %, dry ash-free	51.7	50.9	50.8	50.0	48.7	48.9
H	wt %, dry ash-free	6.1	5.9	5.8	5.9	5.9	5.9
N	wt %, dry ash-free	0.2	0.1	0.3	0.3	0.3	0.2
O as difference	wt %, dry ash-free	42	43	43	44	45	45
Inorganic Content							
K	mg/kg, dry	700	Bdl	2 600	400	7 600	300
Na	mg/kg, dry	200	Bdl	500	100	bdl	bdl
Ca	mg/kg, dry	1 300	Bdl	7 500	800	1 700	100
Mg	mg/kg, dry	200	Bdl	900	100	700	bdl
Si	mg/kg, dry	800	Na	6 000	na	17 000	na
Fe	mg/kg, dry	200	Na	800	na	bdl	na
Al	mg/kg, dry	200	Na	1 200	na	400	na
P	mg/kg, dry	bdl	Na	bdl	na	bdl	na
S	wt %, dry	0.011	0.005	0.029	0.011	0.035	0.008
Cl	wt %, dry	na	0.008	0.159	0.016	0.315	0.017
Lignin and Carbohydrate Content							
total lignin	wt %, dry ash free	30.0	29.1	36.9	31.3	24.5	25.2
Klason lignin	wt %, dry ash-free	29.4	28.8	31.7	27.9	22.6	23.8
acid-soluble lignin	wt %, dry ash-free	0.5	0.4	5.1	3.4	1.9	1.4
polysaccharides	wt %, dry ash-free	52.3	53.5	46.3	47.1	59.6	63.0
extractives	wt %, dry ash-free	1.1	0.7	0.9	0.8	1.1	0.7
Monosaccharide Composition							
rhamnose	wt %, dry ash-free	0.2	0.2	0.4	0.4	0.1	0.1
arabinose	wt %, dry ash-free	1.2	1.0	1.1	0.8	2.8	2.7
galactose	wt %, dry ash-free	2.1	1.9	2.0	2.1	0.9	0.7
glucose	wt %, dry ash-free	39.2	40.7	33.6	35.7	39.8	42.5
xylose	wt %, dry ash-free	7.1	7.0	13.7	12.8	22.7	24.2
mannose	wt %, dry ash-free	8.5	8.8	0.9	0.9	0.3	0.3
fructose	wt %, dry ash-free	bdl	Bdl	bdl	bdl	bdl	bdl
total monosaccharides	wt %, dry ash-free	58.3	59.6	51.8	52.7	66.7	70.7

a For forest and eucalyptus residues: 50 °C, 1% nitric acid, 30 min, B/LL = 1:10. For wheat straw: room temperature, 0.5% nitric acid.

b na = not analyzed, bdl = below detection limit3.1.2 Pilot experiments.

In the case of forest residues and eucalyptus residues, optimal parameters were found to be 50 °C, 1% nitric acid, 30 min leaching time, and a biomass to leaching liquid ratio of 1:10. With wheat straw, the room temperature and 0.5% acid concentration were sufficient, and no significant improvements were obtained at elevated temperatures or higher acid concentrations. These conditions were chosen for the bench-scale experiments and are presented in Table 1. Regarding the mass losses of the leaching experiment on the laboratory scale, there was no significant difference between the acidic and non-acidic conditions. Average mass losses on the dry mass basis with forest residues, eucalyptus residues, and wheat straw were 3.9, 8.0, and 8.1 wt %, respectively.

The effect of acid leaching on the composition of feedstock was further studied by conducting fuel analyses, by analyzing the carbohydrate and lignin contents, and by analyzing the AAEM content of feedstocks before and after leaching for the feedstocks leached under conditions specified in Table 1. All feedstock analyses are presented in Table 2.

Regarding the lignin and carbohydrate content, no major changes were observed between the untreated and leached feedstocks. Fuel analyses indicate that the S and Cl content was lowered and that the volatile content of feedstocks was higher after leaching. Although no dissolution of carbohydrates or lignin was seen, mild acid hydrolysis of lignocellulosic macromolecules may occur^33^ which could explain the slightly higher volatile content in acid-leached feedstocks. In addition, extractive contents decreased after leaching from 1.1 to 0.7 wt % with forest residues and wheat straw. With eucalyptus, loss in extractives was smaller (from 0.9 to 0.8 wt %).

The feedstock analyses, including the elemental composition and metal, before and after leaching are shown in Table 3. The ash content of oat straw was initially particularly high and was greatly reduced after leaching. Based on the results, removal of alkalis was successful, and high removal rates were achieved. A significant amount of potassium was still left in the feedstock due to the high initial concentration. In addition to alkalis, good Cl, S, and P removals and small Fe reduction were achieved. The Si content, on the other hand, increased noticeably. Si is hardly water- or acid-soluble,^8^ so it was not expected to be removed by the leaching procedure conducted in this experiment. Thus, the share of Si in feedstock is increased when other elements are removed.

**Table 3:** Feedstock Analyses for Feedstock Used in Pilot Experiments (Dry Basis)^a^

	unit	wheat straw	oat straw	leached oat straw
moisture	wt %	8.4	16.1	10.0
ash	wt %, dry	6.3	10.2	4.1
volatiles	wt %, dry ash-free	82.1	78.3	81.9
HHV	MJ/kg, dry ash-free	19.68	19.83	20.25
LHV	MJ/kg, dry ash-free	18.35	18.54	18.94
C	wt %, dry ash-free	49.2	49.7	50.3
H	wt %, dry ash-free	6.1	5.9	6.0
N	wt %, dry ash-free	0.5	0.8	0.6
O as difference	wt %, dry ash-free	44	43	43
Inorganic Content				
K	mg/kg, dry	9 900	40 200	2 400
Na	mg/kg, dry	90	1 000	180
Ca	mg/kg, dry	3 600	2 600	610
Mg	mg/kg, dry	700	1 200	130
Si	mg/kg, dry	16 200	11 200	14 600
Fe	mg/kg, dry	240	280	170
Al	mg/kg, dry	Na	64	24
P	mg/kg, dry	520	520	210
S	wt %, dry	0.11	0.756	0.050
Cl	wt %, dry	0.31	0.156	0.075

a na = not analyzed.

### 3.2 Results from Pyrolysis Experiments

#### 3.2.1 Bench-Scale Experiments

Each untreated and leached feedstock was pyrolyzed with the bench-scale BFB unit. Product distributions of successful runs are presented in Table 4. In the case of eucalyptus residues and wheat straw, noticeable increase in organic liquid yield was observed when feedstock was leached. After leaching, organic liquid yield increased 42 and 44% for eucalyptus and wheat straw, respectively. In addition, the amount of char and gases was reduced, and organic liquid yield was improved with leached eucalyptus residues and wheat straw compared to their untreated counterparts. With leached wheat straw, yield of pyrolytic water was also lower, but with leached eucalyptus residues, changes were not significant. In both feedstocks, the AAEM content was reduced greatly with acid leaching. Similar organic liquid yield improvement has been reported by Stefanidis et al.^19^ with high AAEM feedstock, but with feedstock lower in AAEMs, yield improvements have been milder.^12,19^

**Table 4:** Product Distribution from the Bench-Scale Experiments on Dry Basis

		forest residues	eucalyptus residues	leached eucalyptus residues	wheat straw			leached wheat straw
run	unit	1	1	1	1	2	3	1
char	wt %, dry	17.1	23.2	19.3	24.9	29.9	24.6	15.3
pyrolytic gases	wt %, dry	12.3	15.6	7.6	12.1	10.9	11.2	7.6
organic liquid	wt %, dry	54.1	40.8	58.1	42.9	45.3	45.0	63.8
pyrolytic water	wt %, dry	11.9	12.6	11.8	13.4	11.8	11.3	6.9
mass balance closure	wt %, dry	95.4	92.2	96.8	93.4	98.0	92.1	93.6

On the other hand, leached forest residues were challenging to pyrolyze. Some of the biomass was “melted” and agglomerated on the heat carrier, which disturbed feed and temperature control and eventually led to termination of the experiment due to the bed defluidization. The experiment was repeated with a lower feed rate to overcome the observed problems, but even after the adjustments, forest residue particles were not pyrolyzed completely which led to clogging of the equipment. Feed material agglomerated again onto the heat carrier, and cyclones were blocked which resulted in the compromised solid removal. Therefore, no reasonable result was obtained from the pyrolysis experiments of leached forest residues.

Few authors have reported similar bed agglomeration problems with de-ashed feeds.^34−37^ This is interesting since pyrolysis experiments at VTT with low-ash feedstocks have not caused similar problems previously.^22^ Heartwood chips can have the ash content as low as 0.3 wt % (typically 0.3–0.7 wt %) and can be pyrolyzed without problems. However, AAEMs are present at measurable levels in these feedstocks.^38^ Thus, the major difference between our acid-leached forest residues and typical low-ash wood feedstock is that alkali metals were reduced below detection limits in leached forest residues. Based on the thermal degradation studies of pure biomass macromolecules, degradation of hemicellulose and cellulose should occur already at temperatures below 400 °C, but lignin degradation occurs in a much wider temperature range (150–900 °C).^39^ Total absence of catalytically active ash elements can perhaps reduce the cracking of lignocellulosic polymers, especially lignin, so severely that the processability of feedstock is decreased.^37^ Especially, calcium has been reported to be responsible for the cracking of lignin macromolecules into phenolic compounds.^19,22^ Lignin as such is prone to melt and form agglomerates in the fluidized beds.^28,40,41^ Problems with lignin during pyrolysis have been tried to overcome with additives. For example, impregnation of lignin with calcium hydroxide has been reported to help solve these melting problems.^36^

**Figure 3:**
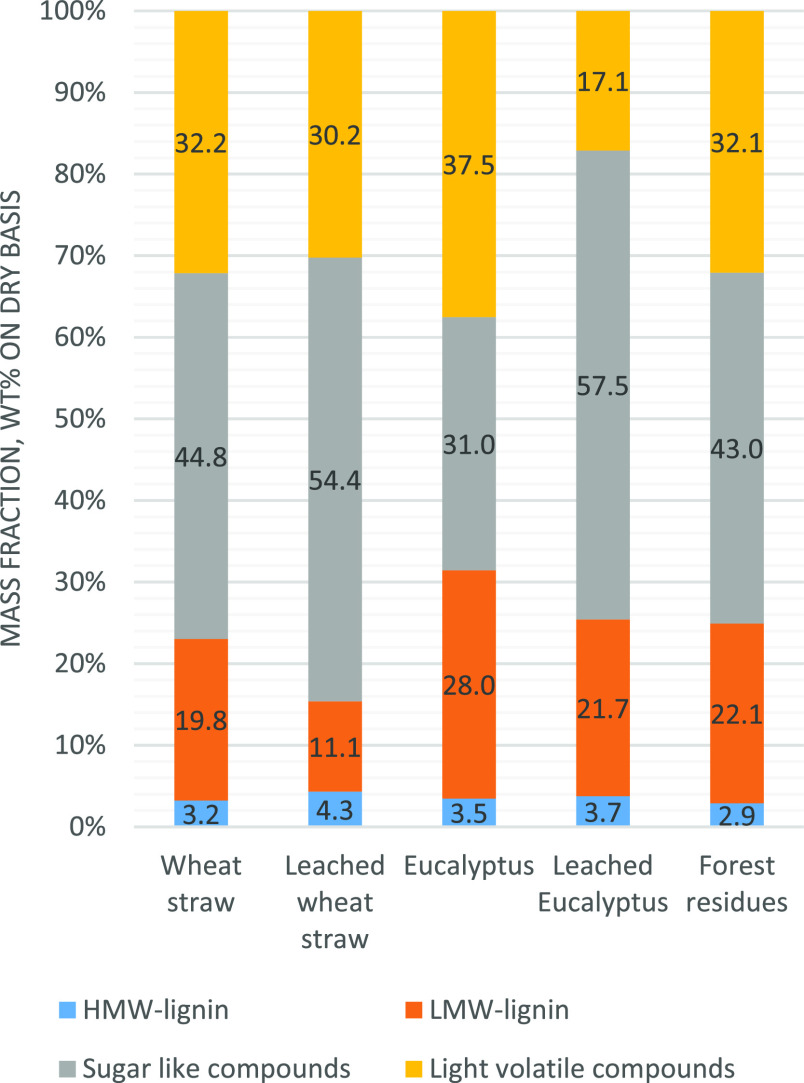
Composition of fast pyrolysis bio-oils produced from untreated and leached feedstocks in the BFB unit on the dry basis.

Chemical compositions of FPBOs are presented in Figure 3. Main difference between the FPBOs produced from leached and untreated feedstocks was the increased portion of the sugar fraction. In addition, slight increase in HMW lignin was observed with leached feedstocks. Variation in light volatile compounds and low-molecular weight lignin (LMW) was also observed. Regarding the chemical composition, results were in line with those presented in the literature.^11,19,42^

Product liquids were also characterized for physical and chemical properties, which are presented in Table 5. Main difference between the liquids from untreated and leached feedstocks was seen in the oxygen content, which was higher in liquids produced from leached feedstocks. The increased oxygen content contributed also to a lower heating value for these liquids. MCR was also higher in FPBOs produced from leached feedstocks. A higher sugar content in these FPBOs is expected to be the reason for a higher MCR and oxygen content. Interestingly, also more carbon monoxide compared to carbon dioxide was formed with the leached feedstocks. With untreated feedstocks, carbon dioxide was more abundant. Cations present in the biomass seem to catalyze more decarboxylation reactions, and when they are removed, decarbonylation reactions become more common. This partly explains the higher oxygen content in leached feedstocks. Non-condensable gas composition is presented in Table 6.

**Table 5:** Physical and Chemical Properties of Fast Pyrolysis Bio-Oils from BFB Experiments on Dry Basis^a^

parameter	unit	forest residues	eucalyptus residues	leached eucalyptus residues	wheat straw	leached wheat straw
water	wt %	22.3	27.5	7.8	21.0	16.4
ash	wt %, dry	0.13	0.04	0.03	0.05	0.04
MCR	wt %, dry	25.1	24.4	31.2	23.7	27.4
C	wt %, dry	57.8	57.5	53.5	54.9	51.0
H	wt %, dry	6.3	6.8	6.2	6.8	6.1
N	wt %, dry	0.1	0.4	0.3	0.3	0.1
O by difference	wt %, dry	36	35	40	38	43
HHV	MJ/kg, dry	24.3	24.1	21.7	22.7	20.4
LHV	MJ/kg, dry	23.0	22.7	20.3	21.2	19.1
pH		2.6	3.1	2.5	2.5	2.3
TAN	mg/KOH/g, dry	85.6	109.1	53.6	115.3	90.1
carbonyls	mmol/g, dry	3.6	4.4	3.9	6.6	7.3
Inorganic Content						
K	mg/kg, dry	bdl	48	bdl	28	bdl
Na	mg/kg, dry	bdl	bdl	bdl	bdl	bdl
Ca	mg/kg, dry	bdl	88	23	6	bdl
Mg	mg/kg, dry	bdl	8	bdl	bdl	bdl
Si	mg/kg, dry	bdl	bdl	bdl	bdl	bdl
S	mg/kg, dry	80	197	128	256	139
Cl	wt %, dry	0.009	0.040	0.023	0.061	0.010

a bdl = below detection limit.

**Table 6:** Composition of Non-condensable Gases from the Bench-Scale Pyrolysis Experiments

compound	unit	forest residues	eucalyptus residues	leached eucalyptus residues	wheat straw	leached wheat straw
hydrogen, H_2_	vol %	3.5	4.0	6.8	0.9	2.3
methane, CH_4_	vol %	8.6	8.0	9.5	3.4	7.4
carbon monoxide, CO	vol %	47.6	38.1	43.2	42.5	55.1
carbon dioxide, CO_2_	vol %	38.6	48.4	39.6	52.0	33.1
ethane, C_2_H_6_	vol %	0.8	0.8	0.8	0.4	0.6
ethylene, C_2_H_4_	vol %	0.9	0.7	0	0.7	1.6
Sum	vol %	100	100	100	100	100

The inorganic content of the FPBOs was also observed to be lower with leached feedstocks. Na was below the detection limit in all samples, but K was reduced below the detection limit with leached eucalyptus and leached wheat straw. Significant reduction in Ca was observed with leached eucalyptus. In addition, the S and Cl content was lower in leached feedstocks. FPBO from forest residues was lowest in all measured inorganics already without leaching, but the feedstock had also the lowest inorganic content at the beginning.

#### 3.2.2 Pilot Experiments

Pilot experiments were carried out with untreated wheat straw and leached oat straw. Utilization of different straw qualities was a suboptimal solution. An optimal solution would have been to use wheat straw also in the pilot-scale leaching experiments, but due to the wheat straw availability issues in Finland at the time of the leaching experiments, oat straw was used. However, based on the feedstock analyses presented in Table 3, major difference between the wheat straw and leached oat straw is the ash and metal content, as CHN and volatiles are at comparable levels.

The target of the experiment with leached oat straw was to run three balance periods with varying temperatures. However, the experiment was terminated prematurely after 13 h due to problems in sand circulation. The reason for problems was identified to be blockages inside the sand pipe, which are expected to be a result of partly melted sticky ash from feedstock. After the failing of the first experiment, few modifications were decided to be made in the system to ensure the success of the second attempt. First, controlled and constant nitrogen feed was added into the sand feeding pipe to make sand more fluidized and to ensure good flow properties of the particles. Second, a metallic mesh was added at the intake of the sand feeding pipe to prevent agglomerated particles to escape the combustor and block the sand feeding pipe of the pyrolysis reactor. Third, the bed sand was kept at 670 °C to reduce the melting of the ash. Finally, the feeding rate was reduced to 15 kg/h from the initially used 18 kg/h. Modification made into the system enabled a successful second attempt.

Similar problems were observed in the experiment with untreated wheat straw, but in this case also, large agglomerates were found from the combustor afterward. With leached oat straw, large agglomerates were not found. However, it is suspected that ash can form sticky or adhesive melt that can glue sand particles together and disturb the sand flow. Straw ash is known to have a low melting point, and it can form agglomerates rather easily at elevated temperatures.^38^ Leaching has been shown to change the melting behavior of greenhouse residue ash,^6^ but the effects of the leaching procedure on thermal behavior of straw ash are still unclear.

Mass balances were calculated for the stable period of operations. Stability was based on the stable reactor temperature and fluidization velocity. Yields are presented in Table 7 for the first (stage 1) and second oat straw experiment (stages 2, 3, and 4) and for the wheat straw experiment.

**Table 7:** Mass Balance from Pilot Test Run Calculated for the Stable Period of Operations as Dry Mass Basis

feedstock	stage	balance period length (h)	feed rate (kg/h)	temperature (°C)	organic liquids (wt %, dry)	pyrolytic water (wt %, dry)	gases (wt %, dry)	char by difference (wt %, dry)
leached oat straw	1	9.1	18	480	55.9	10.2	12.1	21.8
	2	8.5	14.6	488	54.6	12.2	12.0	21.2
	3	8.9	14.7	499	53.1	14.0	13.1	19.8
	4	6.8	17.7	480	53.4	12.9	11.8	21.9
untreated wheat straw	1	33.6	19.6	481	43.6	14.9	14.2	27.3
	2	14.4	19.1	481	43.1	16.8	15.6	24.5
	3	5.6	19.6	462	42.5	13.8	14.6	29.1

**Figure 4:**
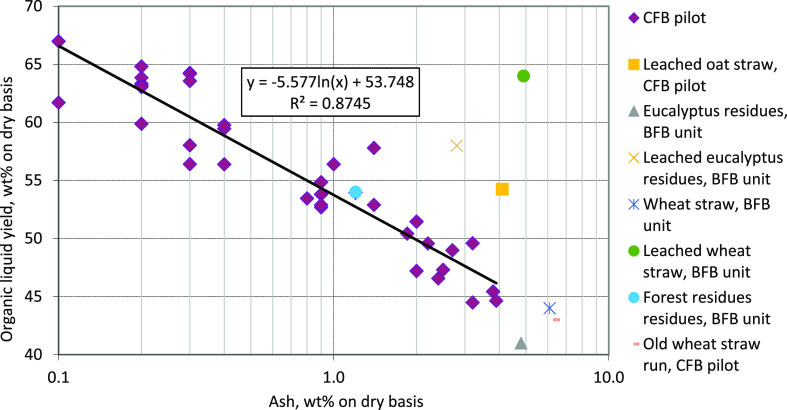
Organic liquid yield as a function of the ash content with different feedstocks from experiments carried out in the VTT’s CFB pilot- and bench-scale BFB unit in the dry mass basis.

**Figure 5:**
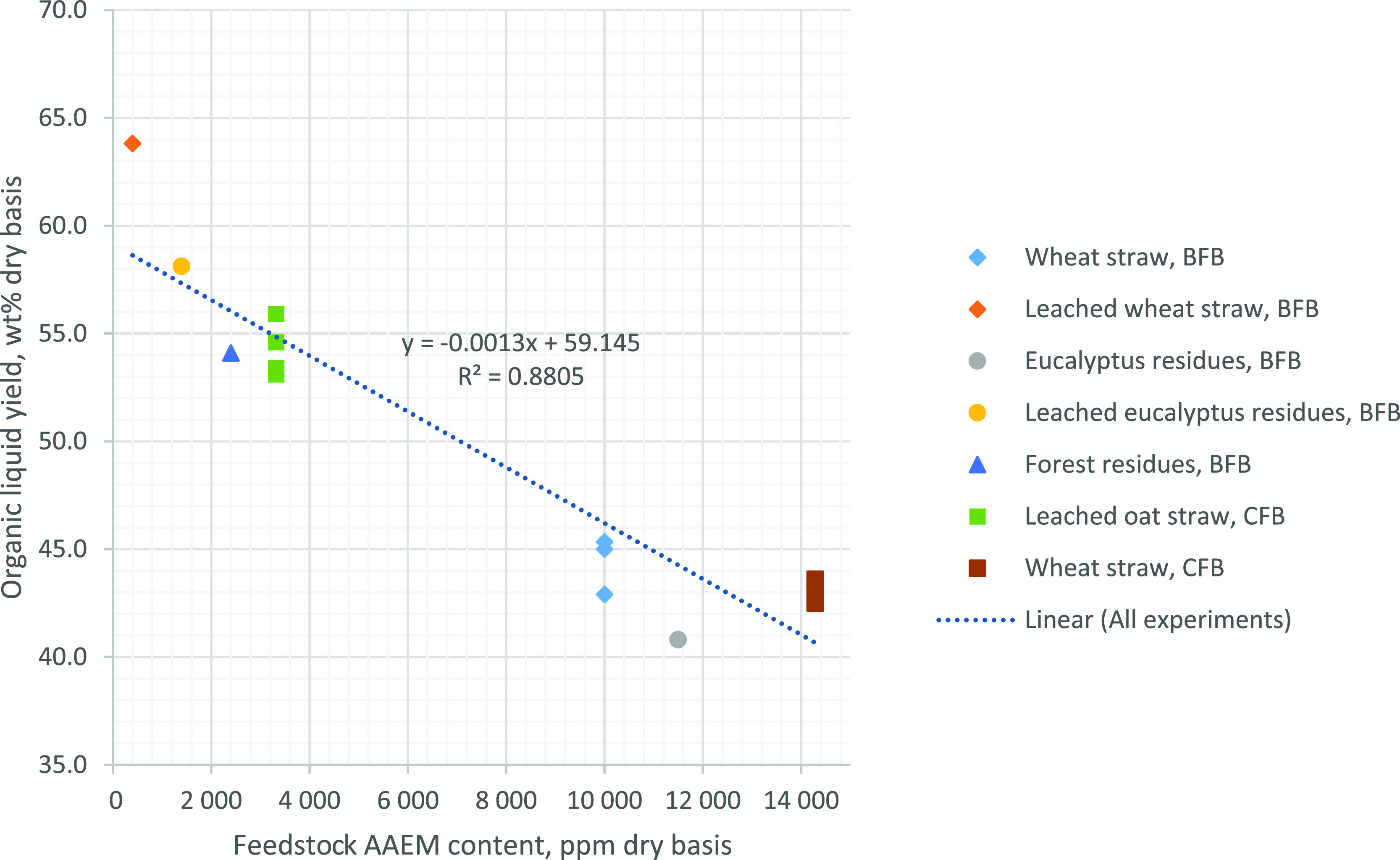
Organic liquid yield as a function of the feedstock AAEM content (K + Ca + Na + Mg) for the feedstocks used in this study.

When the organic liquid yield from leached oat straw is compared with that of wheat straw, the obtained yield from leached oat straw was clearly higher. In addition, when comparison is made with feedstocks containing similar ash levels, leached oat straw seems to give better yields (Figure 4). These results support the conclusion that the concentrations of AAEMs are more important than the total ash content when the organic liquid yield is considered, although the total ash content correlates well with the organic liquid in the case of untreated feedstocks. In fact, when organic liquid yield is plotted against the AAEM content of feedstock, the AAEM content correlates better with organic liquid yield with the feedstocks studied here. Organic liquid yields from current and previous experiments carried out with various feedstock at the VTT’s CFB pilot scale can be seen in Figures 4 and 5.

**Figure 6:**
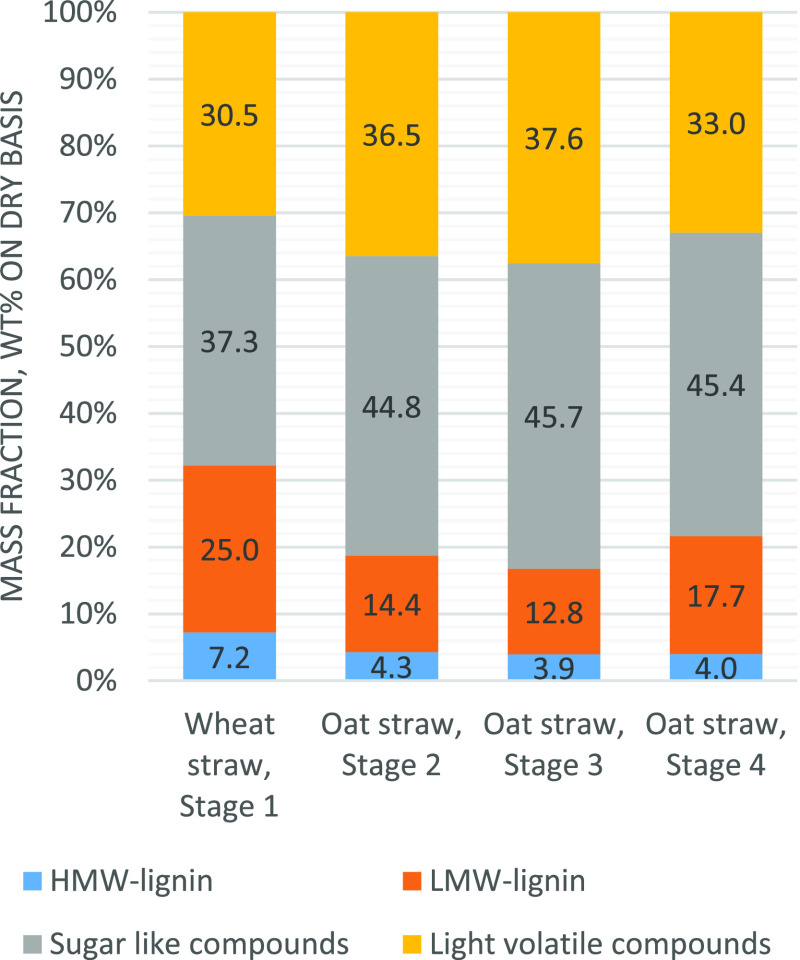
Fast pyrolysis bio-oil composition from the pilot experiments with wheat straw and leached oat straw on the dry basis.

When the composition of produced FPBOs is compared with the composition of liquids produced from untreated wheat straw, results were in line with the bench-scale results and results presented in the literature.^11,19^ Main difference between the FPBOs produced from leached oat straw and FPBOs produced from untreated wheat straw was the increased portion of the sugar fraction in the treated feedstock. The chemical properties, elemental composition, and inorganic content of the produced FPBOs are presented in Table 8, and chemical composition of FPBOs is presented in Figure 6. The average transfer rate of alkalis from leached feedstock to FPBO, calculated from the values presented in Tables 3, 7, and 8, was 20, 25, 12, and 4%, for calcium, magnesium, sodium, and potassium, respectively. Transfer rates were larger than expected from the results presented by Leijenhorst et al.^24^ Leijenhorst et al., however, used rotating cone and screw reactors. A different reactor setup can affect the solid content of the FPBO. Solid removal is critical when low-AAEM content liquids are targeted.^24^

**Table 8:** Characterization of the Produced Fast Pyrolysis Bio-Oils at the CFB Pilot Scale^a^

		wheat straw		leached oat straw	
	unit	stage 1	stage 2	stage 3	stage 4
water	wt %	25.5	26.5	28.9	27.5
Ash	wt %, dry	0.90	0.46	0.42	0.34
MCR	wt %, dry	26.7	27.3	26.9	27.4
C	wt %, dry	56.5	54.7	54.6	55.2
H	wt %, dry	6.9	6.6	6.6	6.5
N	wt %, dry	0.8	0.8	0.7	0.8
Oxygen by difference	wt %, dry	35	38	38	38
Solids	wt %, dry	1.3	0.7	0.7	0.7
TAN	mg KOH/g, dry	84	72.0	73.0	70.8
Inorganic Content					
K	mg/kg, dry	870	88	100	84
Na	mg/kg, dry	bdl	13	28	23
Ca	mg/kg, dry	520	150	130	80
Mg	mg/kg, dry	150	45	35	19
Si	mg/kg, dry	81	960	880	650
S	wt %, dry	0.11	0.09	0.09	0.10
Cl	wt %, dry	0.14	0.03	0.04	0.04

a bdl = below detection limit.

**Figure 7:**
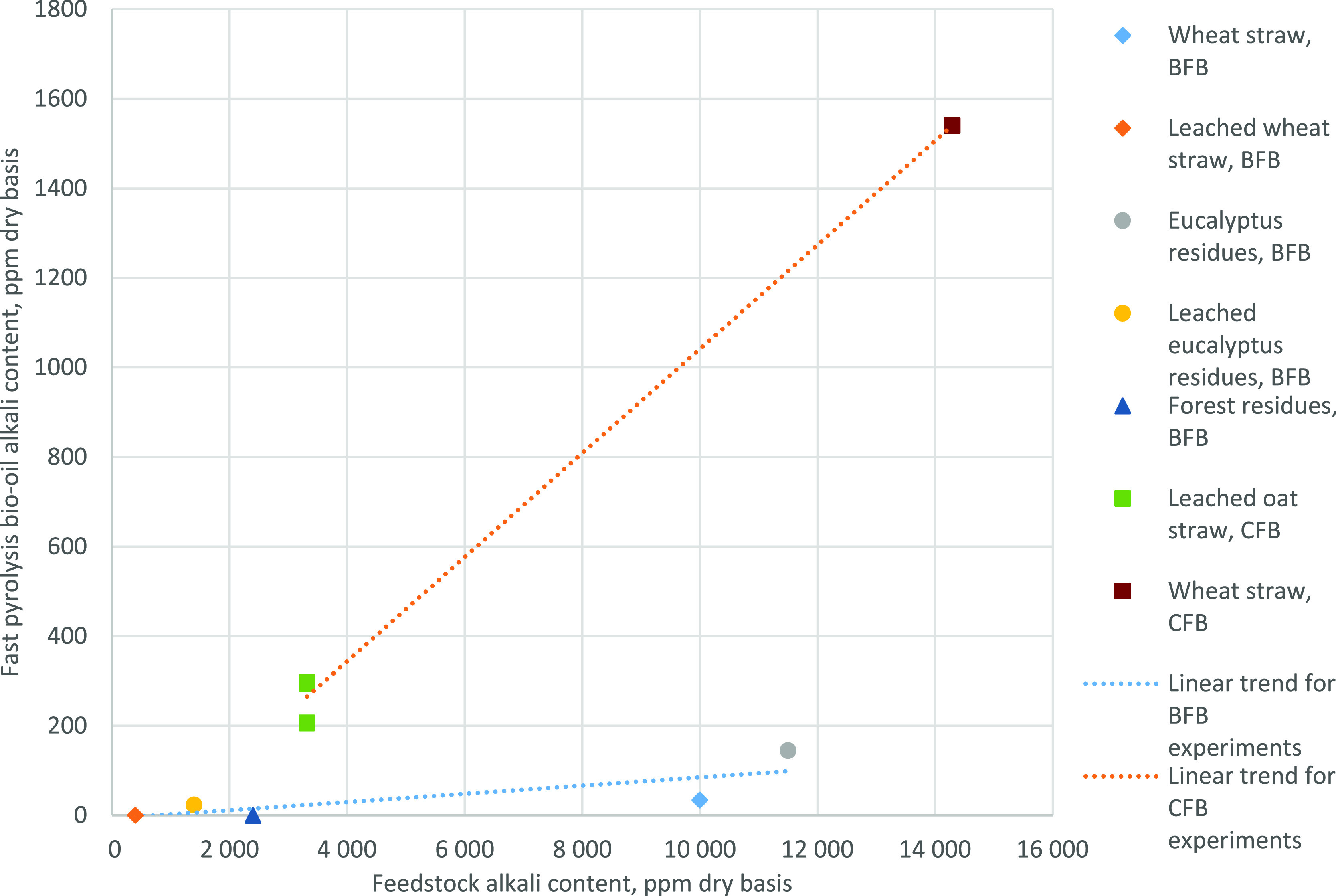
Fast pyrolysis bio-oil AAEM content as a function of the feedstock AAEM content the in the BFB (blue trend line) and CFB (red trend line) units for the feedstocks used in this study.

When the ash and inorganic content of the FPBOs is compared to that of bench-scale BFB experiments, these are higher in FPBOs produced with the CFB pilot (Figure 7). In the bench-scale BFB experiments, solid removal worked well, and the AAEM content of the FPBOs was at a low level, even in the case of high-AAEM feedstocks. In FPBOs produced at the CFB pilot scale, solid and AAEM contents were at a higher level with both feedstocks. Explanation for this can be different cyclone performance in the units. Efficient solid removal is crucial if low-ash and -AAEM FPBOs are the target.^43^

## 4 Conclusions

Various parameters of water and acid treatments of biomass feedstocks were tested in the laboratory- and bench-scale experiments. The total ash content and concentrations of potassium, sodium, calcium, and magnesium were monitored. The most significant leaching parameter was found to be the acidity of the leaching liquid. Untreated and pretreated feedstock was pyrolyzed in bench-scale BFB and pilot-scale CFB units.

Regarding the pyrolysis experiments, significant increase in organic liquid yield was obtained with leached eucalyptus residues and leached wheat straw. Yield correlated well with the feedstock AAEM content. Liquids from leached feedstocks had also a higher sugar and oxygen content compared to their untreated counterparts. In addition, the AAEM, Cl, and S content of the liquid from leached feedstocks was observed to be lower. Interestingly, also more carbon monoxide compared to carbon dioxide was formed with the leached feedstocks. Cations present in the biomass seem to catalyze more decarboxylation reactions, and when they are removed, decarbonylation reactions become more common.

With forest residues, experiments were not successful due to the agglomeration of feed material onto the heat carrier sand and further clogging of the equipment. Total absence of catalytically active ash elements in leached forest residues is suspected to reduce the cracking of lignocellulosic polymers, especially lignin, so severely that the processability of feedstocks is decreased, and melting characteristics of lignin are emphasized. Reactor technology can also have an effect on the FPBO AAEM content, but nevertheless, efficient solid removal is crucial to reach a low AAEM content in FPBO.

Straw and other high-AAEM content feedstocks, where the effects of inorganic removal are emphasized, could be potential feedstock for leaching prior to fast pyrolysis. With sufficiently high yield improvements and quality changes, feedstock pretreatment could be economically beneficial, but this aspect should be considered in more detail in future. Another interesting research question is the effect of these altered qualities on FPBO upgrading.

## Supporting Information Available

Ash and AAEM content from laboratory leaching experiments (PDF)
