# Supplementary material for: Improving Fast Pyrolysis Bio-Oil Yield and Quality by Alkali Removal from Feedstock
Source: Energy Fuels. 2022 Mar 29;36(7):3654–64. doi: 10.1021/acs.energyfuels.1c04331 (PMC8996825; doi:10.1021/acs.energyfuels.1c04331)
Supplement: Supplementary file 1 — ef1c04331_si_001.pdf [file ef1c04331_si_001.pdf]

Supporting Information

Improving fast pyrolysis bio-oil yield and quality by alkali removal from feedstock

Elmeri Pienihäkkinen*, Christian Lindfors, Taina Ohra-aho and Anja Oasmaa

VTT Technical Research Center of Finland Ltd., P.O. Box 1000, FI-02044 VTT, Finland

Corresponding author: Elmeri Pienihäkkinen, elmeri.pienihakkinen@vtt.fi

Table S1. Ash removal in laboratory scale leaching experiment with forest residues feedstock.

Forest residues Sample	Type of water used	T °C	B:LL ratio	Acid wt%	Time min	Ash w-% d.b.	Removal %
Untreated		-	-	-	-	0.74 %	-
1	DI-water	23 °C	1:5	0 %	120	0.52 %	29.74 %
2	DI-water	50 °C	1:5	0 %	120	0.42 %	42.91 %
3	DI-water	80 °C	1:5	0 %	120	0.47 %	36.90 %
4	DI-water	23 °C	1:10	0 %	120	0.43 %	41.36 %
5	DI-water	23 °C	1:20	0 %	120	0.42 %	43.64 %
6	DI-water	23 °C	-	0 %	-	0.62 %	16.28 %
7	DI-water	50 °C	1:10	0 %	120	0.51 %	31.05 %
8	DI-water	50 °C	1:10	0 %	240	0.40 %	45.72 %
9	Tap water	50 °C	1:10	1 %	30	0.30 %	58.96 %
10	DI-water	50 °C	1:5	1 %	30	0.25 %	66.25 %
11	DI-water	50 °C	1:10	1 %	30	0.22 %	70.18 %
12	DI-water	50 °C	1:10	0.5 %	30	0.34 %	53.87 %
13	DI-water	23 °C	1:10	1 %	120	0.15 %	79.76 %
14	DI-water	50 °C	1:10	1 %	120	0.11 %	85.58 %
Bench scale	DI-water	50 °C	1:10	1 %	30	0.29 %	79.76 %

T = temperature, B = biomass, LL = leaching liquid, min = minutes, d.b. = dry basis

Table S2. Ash removal in laboratory scale leaching experiment with eucalyptus residues feedstock.

Eucalyptus residues Sample	Type of water used	T °C	B:LL ratio	Acid wt%	Time min	Ash w-% d.b.	Removal %
Untreated		-	-	-	-	4.75 %	-
1	DI-water	23 °C	1:5	0 %	120	4.03 %	15.23 %
2	DI-water	50 °C	1:5	0 %	120	3.61 %	23.96 %
3	DI-water	80 °C	1:5	0 %	120	3.91 %	17.66 %
4	DI-water	23 °C	1:10	0 %	120	3.75 %	21.01 %
5	DI-water	23 °C	1:20	0 %	120	3.93 %	17.31 %
6	DI-water	23 °C	-	0 %	-	4.36 %	8.31 %
7	DI-water	50 °C	1:10	0 %	120	3.96 %	16.65 %
8	DI-water	50 °C	1:10	0 %	240	3.67 %	22.83 %
9	Tap water	50 °C	1:10	1 %	30	3.12 %	34.31 %
10	DI-water	50 °C	1:5	1 %	30	3.27 %	31.09 %
11	DI-water	50 °C	1:10	1 %	30	2.81 %	40.92 %
12	DI-water	50 °C	1:10	0.5 %	30	3.16 %	33.46 %
13	DI-water	23 °C	1:10	1 %	120	3.14 %	33.93 %
14	DI-water	50 °C	1:10	1 %	120	2.69 %	43.47 %
Bench scale	DI-water	50 °C	1:10	1 %	30	2.54 %	46.46 %

T = temperature, B = biomass, LL = leaching liquid, min = minutes, d.b. = dry basis

Table S3. Ash removal in laboratory scale leaching experiment with wheat straw feedstock.

Wheat straw Sample	Type of water used	T °C	B:LL ratio	Acid wt%	Time min	Ash w-% d.b.	Removal %
Untreated		-	-	-	-	6.91 %	-
1	DI-water	23 °C	1:10	0 %	120	6.12 %	11.37 %
2	DI-water	23 °C	1:20	0 %	120	6.25 %	9.50 %
3	DI-water	50 °C	1:10	0 %	120	6.26 %	9.32 %
4	DI-water	80 °C	1:10	0 %	120	5.78 %	16.36 %
5	DI-water	23 °C	-	0 %	-	6.17 %	10.67 %
6	DI-water	50 °C	1:10	0 %	240	5.86 %	15.20 %
7	Tap water	23 °C	1:10	0.1 %	30	5.82 %	15.77 %
8	DI-water	23 °C	1:10	0.1 %	30	5.77 %	16.42 %
9	DI-water	23 °C	1:10	0.5 %	30	5.70 %	17.39 %
10	DI-water	50 °C	1:10	0.5 %	30	5.67 %	17.82 %
11	DI-water	50 °C	1:10	1 %	30	5.61 %	18.74 %
12	DI-water	23 °C	1:10	1 %	120	5.63 %	18.50 %
13	DI-water	50 °C	1:10	1 %	120	5.72 %	17.24 %
Bench scale	DI-water	23 °C	1:10	0.5 %	30	4.97 %	28.08 %

T = temperature, B = biomass, LL = leaching liquid, min = minutes, d.b. = dry basis

Table S4. AAEM and other inorganic removal in laboratory scale leaching experiment with forest residues feedstock.

**Forest residues** Sample	**Type of water used**	**T °C**	**B:LL ratio**	**Acid wt%**	**Time min**	**K ppm**	**Na ppm**	**Ca ppm**	**Mg ppm**	**Si ppm**	**Fe ppm**	**Al ppm**	**P ppm**	**S ppm**
Untreated	-	-	-	-	-	700	200	1300	200	800	200	200	bdl	200
1	DI-water	23 °C	1:5	0 %	120	100	bdl	1100	200	na	na	na	na	na
2	DI-water	50 °C	1:5	0 %	120	bdl	bdl	1000	200	na	na	na	na	na
3	DI-water	80 °C	1:5	0 %	120	bdl	bdl	700	100	na	na	na	na	na
4	DI-water	23 °C	1:10	0 %	120	100	bdl	900	100	na	na	na	na	na
5	DI-water	23 °C	1:20	0 %	120	bdl	bdl	1000	200	na	na	na	na	na
6	DI-water	23 °C	-	0 %	-	100	bdl	700	bdl	na	na	na	na	na
7	DI-water	50 °C	1:10	0 %	120	bdl	bdl	900	bdl	na	na	na	na	na
8	DI-water	50 °C	1:10	0 %	240	bdl	bdl	900	bdl	na	na	na	na	na
9	Tap water	50 °C	1:10	1 %	30	bdl	100	bdl	bdl	na	na	na	na	na
10	DI-water	50 °C	1:5	1 %	30	bdl	bdl	100	bdl	na	na	na	na	na
11	DI-water	50 °C	1:10	1 %	30	bdl	bdl	100	bdl	na	na	na	na	na
12	DI-water	50 °C	1:10	0.5 %	30	bdl	bdl	100	bdl	na	na	na	na	na
13	DI-water	23 °C	1:10	1 %	120	bdl	bdl	bdl	bdl	na	na	na	na	na
14	DI-water	50 °C	1:10	1 %	120	bdl	bdl	bdl	bdl	na	na	na	na	na
Bench scale	DI-water	50 °C	1:10	1 %	30	bdl	bdl	bdl	bdl	bdl	300	bdl	bdl	100

T = temperature, B = biomass, LL = leaching liquid, min = minutes, d.b. = dry basis, DI = de-ionized, bdl = below detection limit, na = not analyzed

Table S5. AAEM and other inorganic removal in laboratory scale leaching experiment with eucalyptus residue feedstock.

**Eucalyptus residues** Sample	**Type of water used**	**T °C**	**B:LL ratio**	**Acid wt%**	**Time min**	**K ppm**	**Na ppm**	**Ca ppm**	**Mg ppm**	**Si ppm**	**Fe ppm**	**Al ppm**	**P ppm**	**S ppm**
Untreated		-	-	-	-	2600	500	7500	900	6000	800	1200	bdl	400
1	DI-water	23 °C	1:5	0 %	120	600	100	6500	500	na	na	na	na	na
2	DI-water	50 °C	1:5	0 %	120	400	100	6200	500	na	na	na	na	na
3	DI-water	80 °C	1:5	0 %	120	200	bdl	5300	400	na	na	na	na	na
4	DI-water	23 °C	1:10	0 %	120	600	100	5500	500	na	na	na	na	na
5	DI-water	23 °C	1:20	0 %	120	500	100	6300	500	na	na	na	na	na
6	DI-water	23 °C	-	0 %	-	1100	200	4100	200	na	na	na	na	na
7	DI-water	50 °C	1:10	0 %	120	200	bdl	4200	200	na	na	na	na	na
8	DI-water	50 °C	1:10	0 %	240	300	100	5300	300	na	na	na	na	na
9	Tap water	50 °C	1:10	1 %	30	100	100	3400	100	na	na	na	na	na
10	DI-water	50 °C	1:5	1 %	30	100	bdl	4600	100	na	na	na	na	na
11	DI-water	50 °C	1:10	1 %	30	100	bdl	2900	bdl	na	na	na	na	na
12	DI-water	50 °C	1:10	0.5 %	30	200	bdl	4000	100	na	na	na	na	na
13	DI-water	23 °C	1:10	1 %	120	100	bdl	3700	bdl	na	na	na	na	na
14	DI-water	50 °C	1:10	1 %	120	100	bdl	2300	bdl	na	na	na	na	na
Bench scale	DI-water	50 °C	1:10	1 %	30	400	100	800	100	24400	1100	900	bdl	300

T = temperature, B = biomass, LL = leaching liquid, min = minutes, d.b. = dry basis, DI = de-ionized, bdl = below detection limit, na = not analyzed

Table S6. AAEM and other inorganic removal in laboratory scale leaching experiment with wheat straw feedstock.

**Wheat straw** Sample	**Type of water used**	**T °C**	**B:LL ratio**	**Acid wt%**	**Time min**	**K ppm**	**Na ppm**	**Ca ppm**	**Mg ppm**	**Si ppm**	**Fe ppm**	**Al ppm**	**P ppm**	**S ppm**
Untreated		-	-	-	-	7600	bdl	1700	700	17000	bdl	400	bdl	400
1	DI-water	23 °C	1:10	0 %	120	1000	bdl	900	100	na	na	na	na	na
2	DI-water	23 °C	1:20	0 %	120	1000	bdl	1100	100	na	na	na	na	na
3	DI-water	50 °C	1:10	0 %	120	900	bdl	1100	100	na	na	na	na	na
4	DI-water	80 °C	1:10	0 %	120	800	200	1100	100	na	na	na	na	na
5	DI-water	23 °C	-	0 %	-	1400	200	1200	200	na	na	na	na	na
6	DI-water	50 °C	1:10	0 %	240	700	bdl	1000	100	na	na	na	na	na
7	Tap water	23 °C	1:10	0.1 %	30	300	100	1000	100	na	na	na	na	na
8	DI-water	23 °C	1:10	0.1 %	30	300	bdl	600	100	na	na	na	na	na
9	DI-water	23 °C	1:10	0.5 %	30	300	bdl	200	100	na	na	na	na	na
10	DI-water	50 °C	1:10	0.5 %	30	300	bdl	100	100	na	na	na	na	na
11	DI-water	50 °C	1:10	1 %	30	300	bdl	100	100	na	na	na	na	na
12	DI-water	23 °C	1:10	1 %	120	400	bdl	bdl	bdl	na	na	na	na	na
13	DI-water	50 °C	1:10	1 %	120	200	bdl	bdl	bdl	na	na	na	na	na
Bench scale	DI-water	23 °C	1:10	0.5 %	30	300	bdl	100	bdl	25000	bdl	bdl	bdl	100

T = temperature, B = biomass, LL = leaching liquid, min = minutes, d.b. = dry basis, DI = de-ionized, bdl = below detection limit, na = not analyzed
